# Synthesis of fused uracils: pyrano[2,3-*d*]pyrimidines and 1,4-bis(pyrano[2,3-*d*]pyrimidinyl)benzenes by domino Knoevenagel/Diels-Alder reactions

**DOI:** 10.1007/s00706-012-0781-x

**Published:** 2012-05-24

**Authors:** Aleksandra Pałasz

**Affiliations:** Department of Organic Chemistry, Jagiellonian University, Kraków, Poland

**Keywords:** Cycloadditions, Drug research, Michael addition, One-pot synthesis

## Abstract

**Abstract:**

Knoevenagel condensation of barbituric acids with aromatic aldehydes containing one or two formyl groups was carried out. 5-Arylidenebarbituric acids underwent smooth hetero-Diels-Alder (HDA) reactions with enol ethers to afford *cis* and *trans* diastereoisomers of pyrano[2,3-*d*]pyrimidine-2,4-diones and 5,5′-(1,4-phenylene)bis[2*H*-pyrano[2,3-*d*]pyrimidine-2,4(3*H*)-dione] derivatives in excellent yields (75–88 %). Syntheses were realized by Knoevenagel condensation and HDA reaction in four different reaction conditions: Knoevenagel condensation in water and Diels-Alder reaction in methylene chloride solution, Knoevenagel condensation in water and Diels-Alder reaction without solvent, three-component one-pot reaction in methylene chloride solution, or three-component one-pot reaction in water. All reactions were carried out without catalyst at room temperature. The reactions of malononitrile with Knoevenagel condensation products of barbituric acids and heteroaromatic aldehydes or terephthalaldehyde were examined and did not provide corresponding pyranopyrimidines.

**Graphical Abstract:**

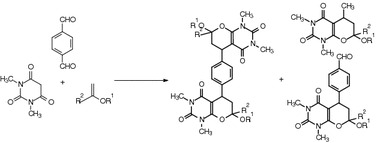
.

## Introduction

Pyran derivatives are common structural subunits in a variety of important natural products, including carbohydrates, alkaloids, polyether antibiotics, pheromones, and iridoids [1, 2]. Uracil is one of the five nucleobases and therefore an important component of nucleic acids. Uracil and its fused derivatives, such as pyrano[2,3-*d*]pyrimidines, pyrido[2,3-*d*]pyrimidines, pyrazo[3,4-*d*]pyrimidines, or pyrimido[4,5-*d*]pyrimidines, are reported to have a wide range of biological activities such as antiallergic [3], antihypertensive [4], cardiotonic [5], bronchiodilator [6], antibronchitic [7], or antitumor [8] activity. The preparation of the compounds containing a pyran and an uracil ring poses significant synthetic challenges. 3,4-Dihydro-2*H*-pyrans can be efficiently synthesized by inverse-electron-demand hetero-Diels-Alder (HDA) reactions of α,β-unsaturated carbonyl compounds representing an 1-oxa-1,3-butadiene system with enol ethers [9–11]. It has been stated that introducing an electron withdrawing group in the 1-oxa-1,3-diene systems can enhance their reactivity [12–15]. In our recent work, we showed that intermolecular and intramolecular HDA reactions are a powerful tool in the synthesis of 2*H*-pyran and polycyclic 2*H*-pyran derivatives [16–24]. Also recently, as a continuation of the investigations of organic reactions performed in aqueous medium, a green approach to the synthesis of fused uracils 2-thioxopyrano[2,3-*d*]pyrimidin-4-ones and pyrano[2,3-*d*]pyrimidin-2,4-diones was made. Three-component one-pot syntheses of annulated uracils were performed in aqueous suspensions by domino Knoevenagel/Diels-Alder reactions without a catalyst and at room temperature [25]. In our last work we also investigated inverse-electron demand Diels-Alder cycloadditions of sterically hindered cycloalkylidene derivatives of benzoyl acetonitrile and *N*,*N*′-dimethylbarbituric acid with enol ethers, cyclic enol ethers, and also sterically hindered cycloalkylidenecycloalkanes [26]. Fused spirouracils and fused dispirouracils can be obtained by this method.

The same α,β-unsaturated carbonyl compounds, obtained by Knoevenagel condensation of the appropriate CH acids and aromatic aldehydes, can be used as substrates in pyran synthesis by conjugate addition-cyclization with malononitrile or cyanoacetate [27–29]. Pyrano[2,3-*d*]pyrimidine derivatives can be prepared by conjugate addition-cyclization of malononitrile to 5-arylidenebarbituric acids, or general procedures include the reaction of arylidenemalononitriles with barbituric acids under traditional hot reaction conditions [30, 31] or under microwave irradiation [32]. Recently, the synthesis of pyrano[2,3-*d*]pyrimidines by simply ball-milling a stoichiometric mixture of an aldehyde, malononitrile, and barbituric acids without any catalyst or solvent was described [33]. Also microwave-assisted three-component cyclocondensation of aldehydes, malononitrile, and barbituric acids proceeds in the absence or presence of triethylamine to afford pyrano[2,3-*d*]pyrimidines [34]. Direct condensation of aldehydes, malononitrile, and barbituric acids in aqueous media has been reported under heating [35] or under ultrasound irradiation [36].

Therefore, 5-arylidene derivatives of barbituric acids seem to be excellent intermediates in pyran synthesis both by HDA reaction and by conjugate addition-cyclization.

## Results and discussion

The main aim of the studies was the synthesis of new (1,4-phenylene)bis[2*H*-pyrano[2,3-*d*]pyrimidine-2,4(3*H*)-dione] derivatives containing two fused uracil moieties joined by a benzene ring. Syntheses were realized by Knoevenagel condensation and HDA reaction in four different reaction conditions: A—Knoevenagel condensation in water and HDA reaction in methylene chloride as solvent, B—Knoevenagel condensation in water and HDA reaction without solvent, C—three-component one-pot reaction in methylene chloride as solvent, and D—three-component one-pot reaction in water. All the reactions were carried out at room temperature in the absence of catalyst.

**Scheme 1:**
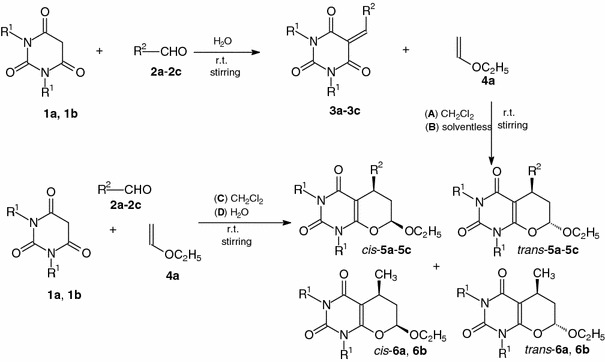

First, procedures A–D were examined for the Knoevenagel condensation of barbituric acids with aromatic aldehydes containing only one formyl group and HDA reactions with enol ether. 5-Arylidenebarbituric acids **3a**–**3c**, as potential heterodienes in Diels-Alder reactions, were synthesized by condensations of *N*,*N*′-dimethylbarbituric acid (**1a**) or barbituric acid (**1b**) with aromatic aldehydes **2a**–**2c** in water without catalyst and at room temperature according the procedure described in the literature [37] (Scheme 1). The condensations occurred smoothly and were completed in just an hour, giving excellent yields (95–98 %) of Knoevenagel products **3a**–**3c**. The cycloaddition reactions of **3a**–**3c** with a tenfold excess of ethyl vinyl ether **4** were performed with methylene chloride as the solvent (conditions A) or in the absence of solvent (conditions B) at room temperature for the time given in Table 1. New 2*H*-pyrano[2,3-*d*]pyrimidine-2,4(3*H*)-diones **5a**–**5c** were obtained in 77–88 % yields (Scheme 1; Table 1). Next, three-component one-pot synthesis of uracils **5a**–**5c** by domino Knoevenagel/Diels-Alder reactions was investigated in methylene chloride (conditions C) or in aqueous medium (conditions D). The experimental procedure was simple: equimolar amounts of barbituric acid **1a** or **1b** and aromatic aldehyde **2a**–**2c** were mixed with a tenfold excess of enol ether **4** in methylene chloride (conditions C) or in aqueous medium (conditions D) (Scheme 1; Table 1). The progress of the reactions was monitored by TLC. The ratios of the *cis*/*trans* diastereoisomers of the pyrano[2,3-*d*]pyrimidine-2,4-diones **5a**–**5c** were determined on the basis of ^1^H NMR spectra of the crude products, analyzing the signals of protons 5-H and 7-H. The unexpected 5-methyl-substituted derivatives of pyrano[2,3-*d*]pyrimidines **6a**–**6b** were obtained in aqueous medium (conditions D). This was determined on the basis of the ^1^H NMR spectra of the crude products. Formation of these compounds can be explained as the result the three-component reaction of barbituric acid **1a** or **1b**, the in situ generated acetaldehyde and ethyl-vinyl ether **4**. The addition of water to ether **4** catalyzed by barbituric acid provides a hemiacetal, which undergoes ethanol elimination to produce the enol tautomer or finally keto tautomer of acetaldehyde. Only compounds *cis*-**6a** and *trans*-**6a** were separated in small amounts by column chromatography.

**Table 1:** Synthesis of the cycloadducts **5a**–**5c** by Knoevenagel condensation and HDA reaction in the reaction conditions A–D

Entry	Method	**1**	R^1^	**2**	R^2^	**3**	**5**	**6**	Reaction time/h	Yield/% of **5** ^a^	Ratio of *cis*-**5**/ *trans*-**5** ^b^
1	A	**1a**	CH_3_	**2a**	4-BrC_6_H_4_	**3a**	**5a**	**–**	15	87	1.8:1
2	B	**1a**	CH_3_	**2a**	4-BrC_6_H_4_	**3a**	**5a**	–	12	86	1.6:1
3	C	**1a**	CH_3_	**2a**	4-BrC_6_H_4_	**–**	**5a**	–	15	84	2.5:1
4	D	**1a**	CH_3_	**2a**	4-BrC_6_H_4_	**–**	**5a**	**6a**	7	82	7.2:1
5	A	**1a**	CH_3_	**2b**	4-ClC_6_H_4_	**3b**	**5b**	**–**	13	81	2.3:1
6	B	**1a**	CH_3_	**2b**	4-ClC_6_H_4_	**3b**	**5b**	–	12	82	1.8:1
7	C	**1a**	CH_3_	**2b**	4-ClC_6_H_4_	**–**	**5b**	–	13	87	2.5:1
8	D	**1a**	CH_3_	**2b**	4-ClC_6_H_4_	**–**	**5b**	**6a**	6	86	6.9:1
9	A	**1b**	H	**2c**	4-H_3_COC_6_H_4_	**3c**	**5c**	**–**	24	86	2.0:1
10	B	**1b**	H	**2c**	4-H_3_COC_6_H_4_	**3c**	**5c**	–	20	77	1.5:1
11	C	**1b**	H	**2c**	4-H_3_COC_6_H_4_	**–**	**5c**	–	22	82	2.2:1
12	D	**1b**	H	**2c**	4-H_3_COC_6_H_4_	**–**	**5c**	**6b**	12	88	5.6:1

^a^Isolated yields after column chromatography

^b^Ratio based on ^1^H NMR (300 MHz) spectra of crude products

All diastereoisomers of compounds **5a**–**5c** were very easily separated by column chromatography using *t*-butyl methyl ether as an eluent because the difference between *R*_f_ (*cis*) and *R*_f_ (*trans*) was approximately 0.2. Cycloadducts *cis*-**5a**–**5c** were the major products in all reactions. Three-component one-pot syntheses of pyrano[2,3-*d*]pyrimidines performed in aqueous medium (conditions D) were faster than those executed in dichloromethane or under solvent-free conditions, and *cis*/*trans* selectivity was significantly improved.

**Scheme 2:**
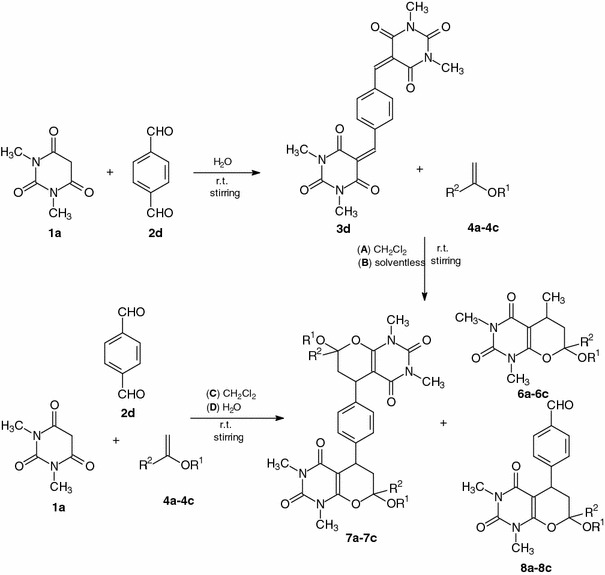

In the second step of the studies, it was decided to test the synthetic approach to the Knoevenagel condensation of barbituric acid with an aromatic aldehyde containing two formyl groups, terephthalaldehyde. HDA reactions with enol ether were performed in conditions A–D. Condensation of *N*,*N*′-dimethylbarbituric acid with terephthalaldehyde (**2d**) was carried out in water without catalyst and at room temperature, giving Knoevenagel product **3d** with 97 % yield after 1 h (Scheme 2). It is worth noting that there is only one synthetic method for this compound described in the literature [38], but it required drastic conditions, with acetic acid and sulfuric acid as the reactive media. The cycloaddition reactions of **3d** with a tenfold excess of enol ethers **4a**–**4c** were performed with methylene chloride as the solvent (conditions A) or in the absence of solvent (conditions B) at room temperature for the time given in Table 2. Also three-component one-pot syntheses of compounds **7a**–**7c** by domino Knoevenagel/Diels-Alder reactions were investigated in conditions C and D. Equimolar amounts of *N*,*N*′-dimethylbarbituric acid and 1,4-benzenedicarbaldehyde were mixed with a tenfold excess of enol ethers **4a**–**4c** in methylene chloride (conditions C) or in aqueous medium (conditions D) (Scheme 2; Table 2). 5,5′-(1,4-Phenylene)bis[2*H*-pyrano[2,3-*d*]pyrimidine-2,4(3*H*)-dione] derivatives **7a**–**7c** were obtained in 75–82 % yields. The progress of the reactions was monitored by TLC. The ratios of the *cis*/*trans* diastereoisomers of cycloadducts **7a**–**7c** were determined on the basis of ^1^H NMR spectra of crude products, analyzing the signals of protons 5-H and 7-H. Cycloadducts *cis*-**7a**–**7c** were the major products. The unexpected pyrano[2,3-*d*]pyrimidines **6a**–**6c** (conditions D) and **8a**–**8c** (conditions C, D) were also obtained in small amounts. It was determined on the basis of the ^1^H NMR spectra of the crude products. Formation of compounds **6a**–**6c** was explained above. Cycloadducts **8a**–**8c** were obtained as the result of Knoevenagel reaction of barbituric acid **1a** and only one formyl group of dicarbaldehyde **2d**. Only compounds *cis*-**6a**, *trans*-**6a**, *cis*-**8a**, and *trans*-**8a** were isolated by column chromatography.

**Table 2:** Synthesis of the cycloadducts **7a**–**7c** by Knoevenagel condensation and HDA reaction in the reaction conditions A–D

Entry	Method	**1**	**2**	**3**	**4**	R^1^	R^2^	**6**	**7**	**8**	Reaction time/h	Yield/% of **7** ^a^	Ratio of *cis*-**7**/ *trans*-**7** ^b^
1	A	**1a**	**2d**	**3d**	**4a**	C_2_H_5_	H	**–**	**7a**	**8a**	18	82	>100:1
2	B	**1a**	**2d**	**3d**	**4a**	C_2_H_5_	H	–	**7a**	**8a**	16	81	>100:1
3	C	**1a**	**2d**	**–**	**4a**	C_2_H_5_	H	–	**7a**	**8a**	15	80	>100:1
4	D	**1a**	**2d**	**–**	**4a**	C_2_H_5_	H	**6a**	**7a**	**8a**	8	82	>100:1
5	A	**1a**	**2d**	**3d**	**4b**	i-Bu	H	**–**	**7b**	**8b**	18	81	6.3:1
6	B	**1a**	**2d**	**3d**	**4b**	i-Bu	H	–	**7b**	**8b**	17	78	5.9:1
7	C	**1a**	**2d**	**–**	**4b**	i-Bu	H	–	**7b**	**8b**	15	77	6.5:1
8	D	**1a**	**2d**	**–**	**4b**	i-Bu	H	**6b**	**7b**	**8b**	8	76	8.1:1
9	A	**1a**	**2d**	**3d**	**4c**	CH_3_	CH_3_	**–**	**7c**	**8c**	24	78	5.3:1
10	B	**1a**	**2d**	**3d**	**4c**	CH_3_	CH_3_	–	**7c**	**8c**	20	75	5.5:1
11	C	**1a**	**2d**	**–**	**4c**	CH_3_	CH_3_	–	**7c**	**8c**	18	82	5.2:1
12	D	**1a**	**2d**	**–**	**4c**	CH_3_	CH_3_	**6c**	**7c**	**8c**	10	80	7.5:1

^a^Isolated yields after column chromatography

^b^Ratio based on ^1^H NMR (300 MHz) spectra of crude products

The three-component one-pot syntheses of pyrano[2,3-*d*]pyrimidines **7a**–**7c** performed in aqueous medium (condition D) were faster than those executed in dichloromethane or under solvent-free conditions, and *cis*/*trans* selectivity was the highest for these reactions.

Compounds **5a**–**5c**, **6a**, **7a**–**7c**, and **8a** were characterized by ^1^H, ^13^C NMR, IR, and elemental analysis. ^1^H and ^13^C signal assignments were confirmed by two-dimensional COSY and HETCOR NMR spectra. The relative *cis* and *trans* configuration of the C-5, C-7 substituents were assigned on the basis of ^1^H NMR spectra. They were deduced from the chemical shift values and coupling constants of the protons attached to C-5 and C-7 of the dihydropyran ring that exists in a half-chair conformation (Table 3).

**Table 3:** Signals of proton 5-H and 7-H in ^1^H NMR spectra of products **5a**–**5c**, **6a**, **7a**–**7c**, and **8a**

Compound	dd 5-H δ/ppm *J*_6ax,5_/*J*_6eq,5_/Hz	dd 7-H δ/ppm *J*_6ax,7_/*J*_6eq,7_/Hz	Compound	dd 5-H δ/ppm *J*_6ax,5_/*J*_6eq,5_/Hz	dd 7-H δ/ppm *J*_6ax,7_/*J*_6eq,7_/Hz
*cis*-**5a**	4.00 7.5/5.1	5.38 4.8/2.7	*trans*-**5a**	4.11 5.7/5.4	5.17 7.5/2.4
*cis*-**5b**	4.02 7.5/5.1	5.38 4.5/2.7	*trans*-**5b**	4.12 5.7/5.1	5.17 7.8/2.7
*cis*-**5c**	3.76 7.2/4.8	5.41 4.5/2.4	*trans*-**5c**	3.81 5.4/4.8	5.08 8.1/2.4
*cis*-**6a**	2.88 ddq 6.9/6.9/3.6	5.40 3.3/3.0	*trans*-**6a**	2.98 ddq 6.9/6.9/3.9	5.30 8.1/2.7
*cis*-**7a**	4.00 7.2/6.3	5.31 5.7/2.7	*trans*-**7a**	–	–
*cis*-**7b**	3.99 7.5/6.0 4.03 6.9/5.4	5.07 6.0/2.4 5.11 8.4/3.0	*trans*-**7b**	4.12 9.3/4.8 4.19 5.4/3.9	5.28 5.7/2.4 5.32 4.2/3.0
*cis*-**7c**	3.97 7.2/5.1	–	*trans*-**7c**	3.93 11.7/6.6	–
*cis*-**8a**	4.13 7.5/5.1	5.42 4.5/2.7	*trans*-**8a**	4.20 6.3/6.0	5.24 6.9/2.4

**Fig. 1:**
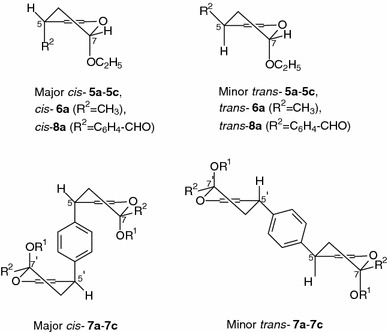
Preferred *cis/trans* configurations and conformations of cycloadducts **5a**–**5c**, **6a**, **7a**–**7c**, and **8a** based on ^1^H NMR analysis

In the ^1^H NMR spectra of the major diastereoisomers *cis*-**5a**–**5c**, *cis*-**6a**, *cis*-**7a**–**7c**, and *cis*-**8a**, the signal of 5-H (5-H and 5′-H for *cis*-**7a**–**7c**) appeared as a doublet of doublets at δ = 3.76–4.13 ppm (for *cis*-**6a**, ddq δ = 2.88 ppm) with coupling constants (^3^ *J* = 6.9–7.5 and 4.8–6.3 Hz) because of coupling with two protons at C-6 (Table 3). Thus, 5-H (5-H and 5′-H for *cis*-**7a**–**7c**) occupies the *pseudo*-*equatorial* position, and the aromatic group adopts the *pseudo*--axial orientation (Fig. 1). The ^1^H NMR spectra of *cis*-**5a**–**5c**, *cis*-**6a**, *cis*-**7a**–**7c**, and *cis*-**8a** reveal the signals of proton 7-H (7-H and 7′-H for *cis*-**7a**–**7c**) as a doublet of doublets at δ = 5.07–5.42 ppm with two small coupling constants ^3^ *J* = 3.3–6.0 Hz (^3^ *J* = 8.4 Hz only for *cis*-**7b**) and 2.4–3.0 Hz. Thus, 7-H (7-H and 7′-H for *cis*-**7a**–**7c**) is in the *equatorial* position, and the alkoxy group occupies the *axial* position (Fig. 1).

For the minor diastereoisomers *trans*-**5a**–**5c**, *trans*-**6a**, *trans*-**7a**–**7c**, and *trans*-**8a**; the protons attached to C-5 (C-5 and C-5′ for *trans*-**7a**–**7c**) give rise to a doublet of doublets with coupling constants ^3^ *J* = 5.4–11.7 and 3.9–6.6 Hz at δ = 3.81–4.20 ppm (for *trans*-**6a**, ddq δ = 2.98 ppm). Thus, 5-H (5-H and 5′-H for *trans*-**7a**–**7c**) is *pseudo*-*axial,* and the *R* ^2^ moiety occupies the *pseudo*-*equatorial* position (Fig. 1). The proton 7-H (7-H and 7′-H for *trans*-**7a**–**7c**) of *trans*-**5a**–**5c**, *trans*-**6a**, *trans*-**7a**–**7c**, and *trans*-**8a** resonates at δ = 5.08–5.32 ppm as a doublet of doublets with two coupling constants (^3^ *J* = 4.2–8.1 and 2.4–3.0 Hz). This suggests that for *trans*-**5a**–**5c**, *trans*-**6a**, *trans*-**7a**–**7c**, and *trans*-**8a**, the conformation with an *axial* alkoxy group is preferred because of stabilization by the anomeric effect (Fig. 1).

**Scheme 3:**
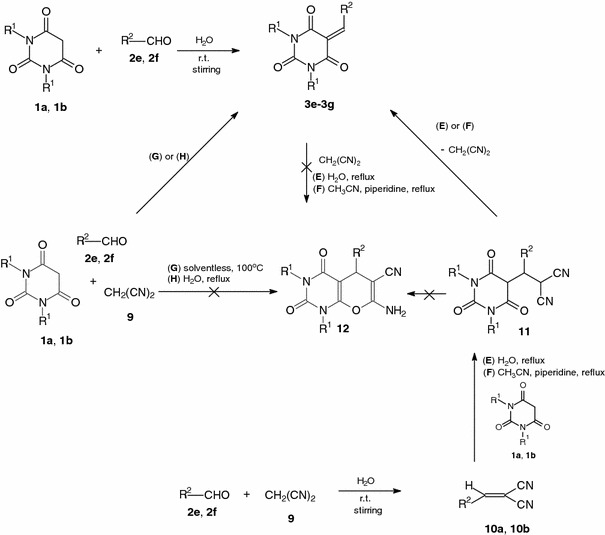

According to the literature, the Knoevenagel condensation products obtained by condensation of barbituric acids and aromatic aldehydes are excellent reagents in pyran synthesis by conjugate addition-cyclization [27–36]. There is no information for the same reactions using heteroaromatic aldehydes or terephthalaldehyde. Therefore, in the next step, the Michael addition-cyclization of malononitrile with α,β-unsaturated carbonyl compounds obtained by Knoevenagel condensation of barbituric acids and heteroaromatic aldehydes or terephthalaldehyde was examined. The reactions of acids **1a**, **1b** with heteroaromatic aldehydes **2e**, **2f** in water at room temperature gave the condensation products **3e** and **3f** with stoichiometric yields after 1 h. Heating of **3e** or **3f** with malononitrile **9** under reflux in water for 1 h (method E, Scheme 3; Table 4, entries 1, 7, 13) or under reflux in acetonitrile in the presence of piperidine for 3 h (method F, Scheme 3; Table 4, entries 2, 8, 14) did not result in compounds **12**.

**Table 4:** Reactions of barbituric acids **1a**, **1b**, heteroaromatic aldehydes **2e**, **2f**, and malononitrile **9** in the reaction conditions E–H

Entry	Reagent	R	Reagent	R	Method	Reagent	R	Product		Yield/% of **3**
								**10**	**3**	
1	**1a**	CH_3_	**2e**	2-Thienyl	E	**9**	**–**	**–**	**3e**	–
2	**1a**	CH_3_	**2e**	2-Thienyl	F	**9**	**–**	**–**	**3e**	–
3	**1a**	CH_3_	**2e**	2-Thienyl	G	**9**	**–**	**–**	**3e**	89
4	**1a**	CH_3_	**2e**	2-Thienyl	H	**9**	**–**	**–**	**3e**	93
5	**2e**	2-Thienyl	**9**	–	E	**1a**	CH_3_	**10a**	**3e**	91
6	**2e**	2-Thienyl	**9**	–	F	**1a**	CH_3_	**10a**	**3e**	85
7	**1b**	H	**2e**	2-Thienyl	E	**9**	–	**–**	**3f**	–
8	**1b**	H	**2e**	2-Thienyl	F	**9**	**–**	**–**	**3f**	**–**
9	**1b**	H	**2e**	2-Thienyl	G	**9**	**–**	**–**	**3f**	85
10	**1b**	H	**2e**	2-Thienyl	H	**9**	–	**–**	**3f**	89
11	**2e**	2-Thienyl	**9**	–	E	**1b**	H	**10a**	**3f**	90
12	**2e**	2-Thienyl	**9**	–	F	**1b**	H	**10a**	**3f**	81
13	**1a**	CH_3_	**2f**	2-Furyl	E	**9**	**–**	**–**	**3g**	–
14	**1a**	CH_3_	**2f**	2-Furyl	F	**9**	**–**	**–**	**3g**	–
15	**1a**	CH_3_	**2f**	2-Furyl	G	**9**	**–**	**–**	**3g**	87
16	**1a**	CH_3_	**2f**	2-Furyl	H	**9**	**–**	**–**	**3g**	90
17	**2f**	2-Furyl	**9**	–	E	**1a**	CH_3_	**10b**	**3g**	88
18	**2f**	2-Furyl	**9**	–	F	**1a**	CH_3_	**10b**	**3g**	79

Therefore, in the next step of the studies, the three-component one-pot reactions of acids **1a**, **1b**, aldehydes **2e**, **2f**, and malononitrile **9** without solvent at 100 °C (method G, Scheme 3; Table 4, entries 3, 9, 15) or in water (method H, Scheme 3; Table 4, entries 4, 10, 16) were examined. There was no trace of the desired products **12** after 1 h of heating, and compounds **3e**–**3g** were obtained in excellent 85–93 % yields as the only products. Therefore, the next attempts to synthesize the compounds **12** were undertaken. Aldehydes **2e**, **2f** were first stirred with malononitrile **9** in water at room temperature, and after 1 h the condensation products **10a** and **10b** were isolated with stoichiometric yields. Further, the mixture of compounds **10a**, **10b** was heated to reflux with barbituric acids **1a** or **1b** in water for 1 h (method E, Scheme 3; Table 4, entries 5, 11, 17) or heated to reflux in acetonitrile in the presence of piperidine for 3 h (method F, Scheme 3; Table 4, entries 6, 12, 18). In these cases also, the only compounds, isolated in good yields of 79–91 % after the reactions, were condensation products **3e**–**3g**. This result suggests that in the first step of the reactions (Table 4, entries 5, 6, 11, 12, 17, 18), the Michael adducts **11** are furnished (Scheme 3). Intermediates **11** did not undergo cyclization with formation of pyrano[2,3-*d*]pyrimidine derivatives **12**, but the elimination of malononitrile led to undesired **3e**–**3g**.

**Scheme 4:**
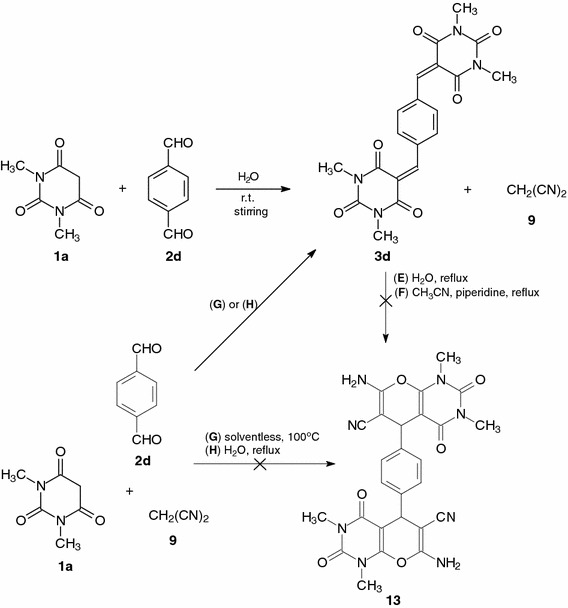

At the end of the study, the reaction procedures E–H presented above were examined for acid **1a**, terephthalaldehyde **2d**, and malononitrile **9**. The reaction of **1a** with aldehyde **2d** in water at room temperature gave condensation product **3d** with almost stoichiometric yield after 1 h. When compound **3d** was heated with malononitrile **9** in water for 1 h (method E, Scheme 4) or in acetonitrile in the presence of piperidine for 3 h (method F, Scheme 4), the expected compound **13** was not obtained.

However, when the three-component one-pot reactions of acid **1a**, aldehyde **2d**, and malononitrile **9** were heated at 100 °C (method G, Scheme 4) without solvent for 1 h or in water under reflux (method H, Scheme 4), compound **3d** was obtained in excellent yield (87–91 %).

In conclusion, new fused uracils of possible pharmacophore, the pyrano[2,3-*d*]pyrimidines and (1,4-phenylene)bis[2*H*-pyrano[2,3-*d*]pyrimidine-2,4(3*H*)-diones], were obtained by domino Knoevenagel/Diels-Alder reactions in different reaction conditions. All reactions were carried out without catalyst and at room temperature. Three-component one-pot syntheses of fused uracils performed in aqueous medium were faster than those executed in dichloromethane or under solventless conditions, and *cis*/*trans* selectivity was the highest for these reactions. The reactions of malononitrile with Knoevenagel condensation products of barbituric acids and heteroaromatic aldehydes or terephthalaldehyde were examined, and they do not provide corresponding pyranopyrimidines. The presented methods avoid the use of catalysts and the heating of reaction mixtures for long times at high temperatures, and the advantages of the presented syntheses are also the excellent yields and short reactions times.

## Experimental

All chemicals were purchased and used without any further purification. The melting points were determined on a Boetius hot stage apparatus. The IR spectra were recorded on a Nicolet IR 200 FT-IR, Thermo Scientific spectrophotometer. NMR spectra were recorded on Bruker Avance II 300 (^1^H: 300.18 MHz, ^13^C: 75.48 MHz) in CDCl3 or DMSO-*d*_6_ with TMS as an internal standard. Microanalyses were performed with a Euro EA 3000 Elemental Analyzer; their results agreed satisfactorily with the calculated values. 5-Arylidenebarbituric acids **3a**–**3g** were obtained according to the general procedure described in Ref. [37].

### *Procedures for the synthesis of pyrano[2,3*-*d]pyrimidine*-*2,4*-*diones****5a*–*5c****,****6a****,****8a****,and5,5′*-*(1,4*-*phenylene)bis[2H*-*pyrano[2,3*-*d]pyrimidine*-*2,4(3H)*-*dione] derivatives****7a*–*7c***

#### Procedure A

A solution of 4.0 mmol **3a**–**3d** (1.29 g **3a**, 1.11 g **3b**, 0.99 g **3c**, 1.64 g **3d**) in dry CH_2_Cl_2_ (50 cm^3^ for **3a**, **3b** and 100 cm^3^ for **3c**, **3d**) and 40 mmol (10 equivalents) of enol ethers **4a**–**4c** (3.8 cm^3^ **4a**, 5.2 cm^3^ **4b**, 3.8 cm^3^ **4c**) was kept at room temperature for the time given in Tables 1 or 2. The progress of the reactions was monitored by TLC. The solvent and excess of ethers were evaporated, and the mixture was separated and purified by column chromatography on silica gel using *t*-butyl methyl ether as an eluent. Recrystallization from a mixture of *t*-butyl methyl ether and petroleum ether gave diastereoisomers **5a**–**5c**, **7a**–**7c** with yields listed in Tables 1 or 2.

#### Procedure B

A mixture of 4.0 mmol of one of the 5-arylidenebarbituric acids **3a**–**3d** (1.29 g **3a**, 1.11 g **3b**, 0.99 g **3c**, 1.64 g **3d**) with a tenfold excess (40 mmol) of one of the enol ethers **4a**–**4c** (3.8 cm^3^ **4a**, 5.2 cm^3^ **4b**, 3.8 cm^3^ **4c**) was stirred without solvent at room temperature for the time given in Tables 1 or 2. The progress of the reactions was monitored by TLC. The excess of ethers was evaporated. Diastereoisomers were separated and recrystallized by the method described in procedure A. Products **5a**–**5c**, **7a**–**7c** were obtained with yields listed in Tables 1 or 2.

#### Procedure C

Equimolar amounts (4.0 mmol) of barbituric acid **1a** (0.625 g) or **1b** (0.51 g) and aldehydes **2a**–**2d** (0.74 g **2a**, 0.56 g **2b**, 0.5 cm^3^ **2c**, 0.27 g (2.0 mmol) **2d**) were mixed with a tenfold excess (40 mmol) of enol ethers **4a**–**4c** (3.8 cm^3^ **4a**, 5.2 cm^3^ **4b**, 3.8 cm^3^ **4c**) in 100 cm^3^ dry CH_2_Cl_2_ at room temperature for the time given in Tables 1 or 2. The progress of the reactions was monitored by TLC. The solvent and excess of ethers were evaporated, and the mixture was separated and purified by the method described in procedure A. Products **5a**–**5c**, **7a**–**7c** were obtained with yields listed in Tables 1 or 2. The diastereoisomers of product **8a** were also separated and recrystallized in small amounts.

#### Procedure D

A suspension of equimolar amounts (4.0 mmol) of barbituric acid **1a** (0.625 g) or **1b** (0.51 g) and appropriate aldehyde **2a**–**2d** (0.74 g **2a**, 0.56 g **2b**, 0.5 cm^3^ **2c**, 0.27 g (2.0 mmol) **2d**) with a tenfold excess (40 mmol) of enol ether **4a**–**4c** (3.8 cm^3^ **4a**, 5.2 cm^3^ **4b**, 3.8 cm^3^ **4c**) in 50 cm^3^ H_2_O was allowed to stay under vigorous stirring at room temperature for the time given in Tables 1 or 2. The progress of the reactions was monitored by TLC. After that, the reaction mixture was extracted with CH_2_Cl_2_. The combined organic layers were dried (MgSO_4_), and the solvent was evaporated under reduced pressure. Diastereoisomers were separated and recrystallized by the method described in procedure A. Products **5a**–**5c**, **7a**–**7c** were obtained with yields listed in Tables 1 or 2. Both diastereoisomers of product **6a** and product **8a** were also separated and recrystallized in small amounts.

##### *(5RS,7SR)*-*5*-*(4*-*Bromophenyl)*-*7*-*ethoxy*-*1,5,6,7*-*tetrahydro*-*1,3*-*dimethyl*-*2H*-*pyrano[2,3*-*d]pyrimidine*-*2,4(3H)*-*dione* (***cis*-5a**, C_17_H_19_BrN_2_O_4_)

Colorless crystals; mp: 169–170 °C; *R*_f_ = 0.48 (*t*-BuOMe); IR (powder): $\bar{\nu }$ = 3,012, 2,926, 1,731, 1,664, 1,504, 1,190, 1,069, 1,017 cm^−1^; ^1^H NMR (300 MHz, CDCl_3_): δ = 1.13 (3H, t, *J* = 7.2 Hz, OCH_2_CH_3_), 2.14 (1H, ddd, *J* = 14.4, 5.1, 4.8 Hz, 6-H), 2.34 (1H, ddd, *J* = 14.1, 7.5, 2.7 Hz, 6-H), 3.28 (3H, s, N-Me), 3.43 (3H, s, N-Me), 3.58 (1H, dq, *J* = 9.3, 6.9 Hz, OCH_2_CH_3_), 3.86 (1H, dq, *J* = 9.3, 6.9 Hz, OCH_2_CH_3_), 4.00 (1H, dd, *J* = 7.5, 5.1 Hz, 5-H), 5.38 (1H, dd, *J* = 4.8, 2.7 Hz, 7-H), 7.07 (2H, d, *J* = 8.4 Hz, Ar), 7.36 (2H, d, *J* = 8.7 Hz, Ar) ppm; ^13^C NMR (75.5 MHz, CDCl_3_): δ = 14.9, 28.0, 28.7, 33.5, 35.4, 65.5, 89.0, 101.9, 119.9, 129.1, 131.1, 142.7, 151.2, 155.1, 162.1 ppm.

##### *(5RS,7RS)*-*5*-*(4*-*Bromophenyl)*-*7*-*ethoxy*-*1,5,6,7*-*tetrahydro*-*1,3*-*dimethyl*-*2H*-*pyrano[2,3*-*d]pyrimidine*-*2,4(3H)*-*dione* (*trans***-5a**, C_17_H_19_BrN_2_O_4_)

Colorless crystals; mp: 198–200 °C; *R*_f_ = 0.65 (*t*-BuOMe); IR (powder): $\bar{\nu }$ = 3,011, 2,964, 1,722, 1,651, 1,503, 1,171, 1,107, 1,017 cm^−1^; ^1^H NMR (300 MHz, CDCl_3_): δ = 1.26 (3H, t, *J* = 7.2 Hz, OCH_2_CH_3_), 2.07 (1H, ddd, *J* = 13.8, 4.8, 2.4 Hz, 6-H), 2.20 (1H, ddd, *J* = 13.8, 7.5, 6.3 Hz, 6-H), 3.29 (3H, s, N-Me), 3.43 (3H, s, N-Me), 3.65 (1H, dq, *J* = 9.3, 6.9 Hz, OCH_2_CH_3_), 3.95 (1H, dq, *J* = 9.3, 6.9 Hz, OCH_2_CH_3_), 4.11 (1H, dd, *J* = 5.7, 5.4 Hz, 5-H), 5.17 (1H, dd, *J* = 7.5, 2.4 Hz, 7-H), 7.06 (2H, d, *J* = 8.4 Hz, Ar), 7.42 (2H, d, *J* = 8.4 Hz, Ar) ppm; ^13^C NMR (75.5 MHz, CDCl_3_): δ = 15.1, 28.0, 28.7, 33.6, 35.9, 66.0, 88.2, 101.2, 120.5, 128.8, 131.8, 142.8, 151.3, 155.4, 162.0 ppm.

##### *(5RS,7SR)*-*5*-*(4*-*Chlorophenyl)*-*7*-*ethoxy*-*1,5,6,7*-*tetrahydro*-*1,3*-*dimethyl*-*2H*-*pyrano[2,3*-*d]pyrimidine*-*2,4(3H)*-*dione* (*cis***-5b**, C_17_H_19_ClN_2_O_4_)

Colorless crystals; mp: 141–142 °C; *R*_f_ = 0.41 (*t*-BuOMe); IR (powder): $\bar{\nu }$ = 2,992, 2,959, 2,887, 1,725, 1,654, 1,503, 1,280, 1,188, 1,046,1,016 cm^−1^; ^1^H NMR (300 MHz, CDCl_3_): δ = 1.13 (3H, t, *J* = 7.2 Hz, OCH_2_CH_3_), 2.14 (1H, ddd, *J* = 14.4, 5.1, 4.8 Hz, 6-H), 2.33 (1H, ddd, *J* = 14.1, 7.5, 2.7 Hz, 6-H), 3.28 (3H, s, N-Me), 3.43 (3H, s, N-Me), 3.58 (1H, dq, *J* = 9.3, 6.9 Hz, OCH_2_CH_3_), 3.86 (1H, dq, *J* = 9.3, 6.9 Hz, OCH_2_CH_3_), 4.02 (1H, dd, *J* = 7.5, 5.1 Hz, 5-H), 5.38 (1H, dd, *J* = 4.5, 2.7 Hz, 7-H), 7.12 (2H, d, *J* = 8.4 Hz, Ar), 7.21 (2H, d, *J* = 8.4 Hz, Ar) ppm; ^13^C NMR (75.5 MHz, CDCl_3_): δ = 14.9, 28.0, 28.7, 33.4, 35.4, 65.5, 89.1, 102.0, 128.2, 128.7, 131.8, 142.2, 151.2, 155.1, 162.1 ppm.

##### *(5RS,7RS)*-*5*-*(4*-*Chlorophenyl)*-*7*-*ethoxy*-*1,5,6,7*-*tetrahydro*-*1,3*-*dimethyl*-*2H*-*pyrano[2,3*-*d]pyrimidine*-*2,4(3H)*-*dione* (*trans***-5b**, C_17_H_19_ClN_2_O_4_)

Colorless crystals; mp: 153–155 °C; *R*_f_ = 0.59 (*t*-BuOMe); IR (powder): $\bar{\nu }$ = 3,004, 2,960, 2,912, 2,887, 1,720, 1,651, 1,505, 1,178, 1,118, 1,035 cm^−1^; ^1^H NMR (300 MHz, CDCl_3_): δ = 1.27 (3H, t, *J* = 6.9 Hz, OCH_2_CH_3_), 2.07 (1H, ddd, *J* = 14.1, 5.1, 2.7 Hz, 6-H), 2.20 (1H, ddd, *J* = 13.8, 7.5, 6.3 Hz, 6-H), 3.29 (3H, s, N-Me), 3.43 (3H, s, N-Me), 3.65 (1H, dq, *J* = 9.3, 6.9 Hz, OCH_2_CH_3_), 3.98 (1H, dq, *J* = 9.3, 6.9 Hz, OCH_2_CH_3_), 4.12 (1H, dd, *J* = 5.7, 5.1 Hz, 5-H), 5.17 (1H, dd, *J* = 7.8, 2.7 Hz, 7-H), 7.11 (2H, d, *J* = 8.4 Hz, Ar), 7.27 (2H, d, *J* = 8.4 Hz, Ar) ppm; ^13^C NMR (75.5 MHz, CDCl_3_): δ = 15.1, 28.0, 28.7, 33.5, 36.0, 66.0, 88.3, 101.4, 128.6, 128.8, 132.4, 142.2, 151.3, 155.4, 162.1 ppm.

##### *(5RS,7SR)*-*7*-*Ethoxy*-*1,5,6,7*-*tetrahydro*-*5*-*(4*-*methoxyphenyl)*-*2H*-*pyrano[2,3*-*d]pyrimidine*-*2,4(3H)*-*dione* (*cis***-5c**, C_16_H_18_N_2_O_5_)

Colorless crystals; mp: 299–300 °C; *R*_f_ = 0.39 (*t*-BuOMe); IR (powder): $\bar{\nu }$ = 3,200, 3,170, 3,012, 2,938, 2,869, 1,732, 1,671, 1,530, 1,270, 1,200, 1,108, 1,068, 1,048 cm^−1^; ^1^H NMR (300 MHz, DMSO-*d*_6_): δ = 1.00 (3H, t, *J* = 7.2 Hz, OCH_2_CH_3_), 1.92 (1H, ddd, *J* = 14.1, 4.8, 4.8 Hz, 6-H), 2.23 (1H, ddd, *J* = 14.1, 7.2, 2.4 Hz, 6-H), 3.50 (1H, dq, *J* = 9.6, 6.9 Hz, OCH_2_CH_3_), 3.69 (3H, s, OCH_3_), 3.71 (1H, dq, *J* = 9.6, 7.2 Hz, OCH_2_CH_3_), 3.76 (1H, dd, *J* = 7.2, 4.8 Hz, 5-H), 5.41 (1H, dd, *J* = 4.5, 2.4 Hz, 7-H), 6.75 (2H, d, *J* = 9.0 Hz, Ar), 7.03 (2H, d, *J* = 8.4 Hz, Ar), 10.69 (1H, s, NH), 11.35 (1H, s, NH) ppm; ^13^C NMR (75.5 MHz, DMSO-*d*_6_): δ = 14.8, 31.4, 35.4, 54.8, 64.1, 87.8, 100.6, 112.8, 128.2, 136.3, 150.0, 156.3, 157.1, 163.4 ppm.

##### *(5RS,7RS)*-*7*-*Ethoxy*-*1,5,6,7*-*tetrahydro*-*5*-*(4*-*methoxyphenyl)*-*2H*-*pyrano[2,3*-*d]pyrimidine*-*2,4(3H)*-*dione* (*trans***-5c**, C_16_H_18_N_2_O_5_)

Colorless crystals; mp: 314–315°C; *R*_f_ = 0.65 (*t*-BuOMe); IR (powder): $\bar{\nu }$ = 3,192, 3,120, 3,003, 2,958, 2,884, 2,851, 1,725, 1,632, 1,529, 1,260, 1,186, 1,088, 1,045 cm^−1^; ^1^H NMR (300 MHz, DMSO-*d*_6_): δ = 1.13 (3H, t, *J* = 7.2 Hz, OCH_2_CH_3_), 1.94 (1H, ddd, *J* = 13.8, 4.5, 2.4 Hz, 6-H), 2.07 (1H, ddd, *J* = 13.8, 8.1, 6.0 Hz, 6-H), 3.61 (1H, dq, *J* = 9.6, 6.9 Hz, OCH_2_CH_3_), 3.71 (3H, s, OCH_3_), 3.81 (1H, dd, *J* = 5.4, 4.8 Hz, 5-H), 3.84 (1H, dq, *J* = 9.6, 6.9 Hz, OCH_2_CH_3_), 5.08 (1H, dd, *J* = 8.1, 2.4 Hz, 7-H), 6.83 (2H, d, *J* = 8.7 Hz, Ar), 7.08 (2H, d, *J* = 8.7 Hz, Ar) 10.72 (1H, s, NH), 11.38 (1H, s, NH) ppm; ^13^C NMR (75.5 MHz, DMSO-*d*_6_): δ = 14.9, 31.8, 36.0, 54.9, 64.8, 87.0, 99.8, 113.6, 128.2, 136.0, 150.1, 156.6, 157.5, 163.4 ppm.

##### *(5RS,7RS)*-*7*-*Ethoxy*-*1,5,6,7*-*tetrahydro*-*1,3,5*-*trimethyl*-*2H*-*pyrano[2,3*-*d]pyrimidine*-*2,4(3H)*-*dione* (*cis***-6a**, C_12_H_18_N_2_O_4_)

Colorless crystals; mp: 79–80 °C; *R*_f_ = 0.37 (*t*-BuOMe); IR (powder): $\bar{\nu }$ = 2,968, 2,934, 2,901, 2,879, 1,701, 1,625, 1,483, 1,183, 1,144, 1,102, 1,022 cm^−1^; ^1^H NMR (300 MHz, CDCl_3_): δ = 1.26 (3H, t, *J* = 7.2 Hz, OCH_2_CH_3_), 1.35 (3H, d, *J* = 6.9 Hz, 5-CH_3_), 1.90 (1H, ddd, *J* = 14.1, 3.6, 3.3 Hz, 6-H), 2.05 (1H, ddd, *J* = 14.1, 6.9, 3.0 Hz, 6-H), 2.88 (1H, ddq, *J* = 6.9, 6.9, 3.6 Hz, 5-H), 3.34 (3H, s, N-Me), 3.36 (3H, s, N-Me), 3.65 (1H, dq, *J* = 9.3, 6.9 Hz, OCH_2_CH_3_), 3.89 (1H, dq, *J* = 9.3, 7.2 Hz, OCH_2_CH_3_), 5.40 (1H, dd, *J* = 3.3, 3.0 Hz, 7-H) ppm; ^13^C NMR (75.5 MHz, CDCl_3_): δ = 15.1, 20.1, 22.6, 27.9, 28.6, 33.4, 65.6, 92.0, 101.8, 151.2, 153.4, 162.7 ppm.

##### *(5RS,7SR)*-*7*-*Ethoxy*-*1,5,6,7*-*tetrahydro*-*1,3,5*-*trimethyl*-*2H*-*pyrano[2,3*-*d]pyrimidine*-*2,4(3H)*-*dione* (*trans-***6a**, C_12_H_18_N_2_O_4_)

Colorless crystals; mp: 88–90 °C; *R*_f_ = 0.43 (*t*-BuOMe); IR (powder): $\bar{\nu }$ = 2,972, 2,931, 2,908, 2,883, 1,701, 1,630, 1,491, 1,182, 1,140, 1,098, 1,020 cm^−1^; ^1^H NMR (300 MHz, CDCl_3_): δ = 1.26 (3H, d, *J* = 6.9 Hz, 5-CH_3_), 1.31 (3H, t, *J* = 7.2 Hz, OCH_2_CH_3_), 1.85 (1H, ddd, *J* = 13.8, 3.9, 2.7 Hz, 6-H), 1.94 (1H, ddd, *J* = 13.8, 8.1, 6.0 Hz, 6-H), 2.98 (1H, ddq, *J* = 6.9, 6.9, 3.9 Hz, 5-H), 3.33 (3H, s, N-Me), 3.35 (3H, s, N-Me), 3.73 (1H, dq, *J* = 9.6, 7.2 Hz, OCH_2_CH_3_), 4.01 (1H, dq, *J* = 9.6, 7.2 Hz, OCH_2_CH_3_), 5.30 (1H, dd, *J* = 8.1, 2.7 Hz, 7-H) ppm; ^13^C NMR (75.5 MHz, CDCl_3_): δ = 15.1, 20.8, 23.2, 27.9, 28.6, 34.6, 66.0, 91.6, 101.2, 151.2, 153.9, 162.6 ppm.

##### *(5RS,7SR,5′RS,7′SR)*-*5,5′*-*(1,4*-*Phenylene)bis[7*-*ethoxy*-*1,5,6,7*-*tetrahydro*-*1,3*-*dimethyl*-*2H*-*pyrano[2,3*-*d]pyrimidine*-*2,4(3H)*-*dione]* (*cis*-**7a**, C_28_H_34_N_4_O_8_)

Colorless crystals; mp: >360 °C; *R*_f_ = 0.14 (*t*-BuOMe); IR (powder): $\bar{\nu }$ = 2,973, 2,926, 2,884, 1,703, 1,635, 1,480, 1,173, 1,132, 1,035, 1,001 cm^−1^; ^1^H NMR (300 MHz, CDCl_3_): δ = 1.15 (3H, t, *J* = 7.2 Hz, OCH_2_CH_3_), 2.17 (2H, ddd, *J* = 14.7, 6.3, 5.7 Hz, 6-H, 6′-H), 2.32 (2H, ddd, *J* = 14.1, 7.2, 2.7 Hz, 6-H, 6′-H), 3.27 (6H, s, N-Me), 3.42 (6H, s, N-Me), 3.58 (2H, dq, *J* = 9.3, 6.9 Hz, OCH_2_CH_3_), 3.86 (2H, dq, *J* = 9.3, 7.2 Hz, OCH_2_CH_3_), 4.00 (2H, dd, *J* = 7.2, 6.3 Hz, 5-H, 5′-H), 5.31 (2H, dd, *J* = 5.7, 2.7 Hz, 7-H, 7′-H), 7.05 (4H, br, Ar) ppm; ^13^C NMR (75.5 MHz, CDCl_3_): δ = 14.9, 27.9, 28.7, 34.0, 36.1, 65.5, 90.0, 102.5, 126.9, 141.1, 151.3, 155.0, 162.1 ppm.

##### *(5RS,7SR,5′RS,7′SR)*-*5,5′*-*(1,4*-*Phenylene)bis[1,5,6,7*-*tetrahydro*-*7*-*isobutoxy*-*1,3*-*dimethyl*-*2H*-*pyrano[2,3*-*d]pyrimidine*-*2,4(3H)*-*dione]* (*cis*-**7b**, C_32_H_42_N_4_O_8_)

Colorless crystals; mp: >360 °C; *R*_f_ = 0.24 (*t*-BuOMe); IR (powder): $\bar{\nu }$ = 2,959, 2,927, 2,864, 2,853, 1,702, 1,636, 1,458, 1,162, 1,154, 1,047, 1,006 cm^−1^; ^1^H NMR (300 MHz, CDCl_3_): δ = 0.90 (12H, d, *J* = 6.6 Hz, OCH_2_CH(CH_3_)_2_), 1.67 (2H, m, OCH_2_CH(CH_3_)_2_), 2.02 (2H, ddd, *J* = 13.8, 4.8, 2.4 Hz, 6-H, 6′-H), 2.20 (2H, ddd, *J* = 13.8, 8.4, 6.0 Hz, 6-H, 6′-H), 3.30 (6H, s, N-Me), 3.41 (6H, s, N-Me), 3.54 (1H, dd, *J* = 9.0, 6.3 Hz, OCH_2_CH(CH_3_)_2_), 3.67 (1H, dd, *J* = 9.0, 6.6 Hz, OCH_2_CH(CH_3_)_2_), 3.99 (1H, dd, *J* = 7.5, 6.0 Hz, 5-H), 4.03 (1H, dd, *J* = 6.9, 5.4 Hz, 5′-H), 5.07 (1H, dd, *J* = 6.0, 2.4 Hz, 7-H), 5.11 (1H, dd, *J* = 8.4, 3.0 Hz, 7′-H), 7.00 (2H, br, Ar), 7.05 (2H, br, Ar) ppm; ^13^C NMR (75.5 MHz, CDCl_3_): δ = 19.0, 19.5, 27.8, 28.3, 28.5, 33.5, 33.7, 36.0, 36.1, 77.2, 88.0, 88.3, 101.8, 102.0, 127.3, 127.5, 141.6, 141.7, 151.3, 155.5, 162.3 ppm.

##### *(5RS,7RS,5′RS,7′RS)*-*5,5′*-*(1,4*-*Phenylene)bis[1,5,6,7*-*tetrahydro*-*7*-*isobutoxy*-*1,3*-*dimethyl*-*2H*-*pyrano[2,3*-*d]pyrimidine*-*2,4(3H)*-*dione]* (*trans*-**7b**, C_32_H_42_N_4_O_8_)

Colorless crystals; mp: >360 °C; *R*_f_ = 0.37 (*t*-BuOMe); IR (powder): $\bar{\nu }$ = 2,955, 2,921, 2,868, 2,851, 1,699, 1,634, 1,455, 1,166, 1,151, 1,053, 1,002 cm^−1^; ^1^H NMR (300 MHz, CDCl_3_): δ = 0.91 (12H, d, *J* = 6.9 Hz, OCH_2_CH(CH_3_)_2_), 1.83 (2H, m, OCH_2_CH(CH_3_)_2_), 2.13 (2H, ddd, *J* = 13.8, 8.7, 3.9 Hz, 6-H, 6′-H), 2.28 (2H, ddd, *J* = 13.8, 5.7, 4.8 Hz, 6-H, 6′-H), 3.29 (6H, s, N-Me), 3.40 (6H, s, N-Me), 3.58 (1H, dd, *J* = 9.0, 6.3 Hz, OCH_2_CH(CH_3_)_2_), 3.65 (1H, dd, *J* = 9.0, 6.6 Hz, OCH_2_CH(CH_3_)_2_), 4.12 (1H, dd, *J* = 9.3, 4.8 Hz, 5-H), 4.19 (1H, dd, *J* = 5.4, 3.9 Hz, 5′-H), 5.28 (1H, dd, *J* = 5.7, 2.4 Hz, 7-H), 5.32 (1H, dd, *J* = 4.2, 3.0 Hz, 7′-H), 7.00 (2H, br, Ar), 7.05 (2H, br, Ar) ppm; ^13^C NMR (75.5 MHz, CDCl_3_): δ = 19.1, 19.2, 28.0, 28.6, 28.7, 33.6, 33.7, 35.4, 35.5, 77.2, 89.2, 90.0, 102.6, 103.0, 126.8, 126.9, 140.8, 141.0, 151.3, 154.9, 162.0 ppm.

##### *(5RS,7SR,5′RS,7′SR)*-*5,5′*-*(1,4*-*Phenylene)bis[1,5,6,7*-*tetrahydro*-*7*-*methoxy*-*1,3,7*-*trimethyl*-*2H*-*pyrano[2,3*-*d]pyrimidine*-*2,4(3H)*-*dione]* (*cis*-**7c**, C_28_H_34_N_4_O_8_)

Colorless crystals; mp: >360 °C; *R*_f_ = 0.27 (*t*-BuOMe); IR (powder): $\bar{\nu }$ = 2984, 2958, 2887, 1700, 1627, 1485, 1455, 1176, 1072, 1042, 1019 cm^−1^; ^1^H NMR (300 MHz, CDCl_3_): δ = 1.53 (3H, s, 7-CH_3_), 1.56 (3H, s, 7′-CH_3_), 2.11 (2H, dd, *J* = 14.1, 7.2 Hz, 6-H, 6′-H), 2.31 (2H, dd, *J* = 14.1, 6.0 Hz, 6-H, 6′-H), 3.19 (6H, s, OCH_3_), 3.29 (6H, s, N-Me), 3.42 (6H, s, N-Me), 3.97 (2H, dd, *J* = 7.2, 5.1 Hz, 5-H), 7.03 (4H, br, Ar) ppm; ^13^C NMR (75.5 MHz, CDCl_3_): δ = 22.3, 27.9, 28.6, 34.3, 39.8, 49.6, 88.9, 105.6, 126.7, 126.9, 140.9, 151.4, 155.2, 162.2 ppm.

##### *(5RS,7RS,5′RS,7′RS)*-*5,5′*-*(1,4*-*Phenylene)bis[1,5,6,7*-*tetrahydro*-*7*-*methoxy*-*1,3,7*-*trimethyl*-*2H*-*pyrano[2,3*-*d]pyrimidine*-*2,4(3H)*-*dione]* (*trans*-**7c**, C_28_H_34_N_4_O_8_)

Colorless crystals; mp: >360 °C; *R*_f_ = 0.39 (*t*-BuOMe); IR (powder): $\bar{\nu }$ = 2,981, 2,952, 2,885, 1,697, 1,631, 1,486, 1,449, 1,172, 1,069, 1,047, 1,018 cm^−1^; ^1^H NMR (300 MHz, CDCl_3_): δ = 1.53 (3H, s, 7-CH_3_), 1.55 (3H, s, 7′-CH_3_), 2.12 (2H, dd, *J* = 14.4, 11.4 Hz, 6-H, 6′-H), 2.33 (2H, dd, *J* = 14.4, 6.9 Hz, 6-H, 6′-H), 3.23 (6H, s, OCH_3_), 3.31 (6H, s, N-Me), 3.42 (6H, s, N-Me), 3.93 (2H, dd, *J* = 11.7, 6.6 Hz, 5-H, 5′-H), 7.02 (4H, br, Ar) ppm; ^13^C NMR (75.5 MHz, CDCl_3_): δ = 22.1, 27.8, 28.5, 34.2, 43.3, 50.0, 91.2, 104.0, 126.5, 127.2, 141.6, 151.4, 154.6, 161.8 ppm.

##### *(5RS,7SR)*-*7*-*Ethoxy*-*5*-*(4*-*formylphenyl)*-*1,5,6,7*-*tetrahydro*-*1,3*-*dimethyl*-*2H*-*pyrano[2,3*-*d]pyrimidine*-*2,4(3H)*-*dione* (*cis***-8a**, C_18_H_20_N_2_O_5_)

Colorless crystals; mp: 335–337 °C; *R*_f_ = 0.19 (*t*-BuOMe); IR (powder): $\bar{\nu }$ = 2,975, 2,937, 2,898, 1,703, 1,634, 1,571, 1,486, 1,379, 1,170, 1,092, 1,004 cm^−1^; ^1^H NMR (300 MHz, CDCl_3_): δ = 1.26 (3H, t, *J* = 7.2 Hz, OCH_2_CH_3_), 2.22 (1H, ddd, *J* = 14.1, 4.8, 4.5 Hz, 6-H), 2.38 (1H, ddd, *J* = 14.4, 7.5, 2.7 Hz, 6-H), 3.28 (3H, s, N-Me), 3.45 (3H, s, N-Me), 3.57 (1H, dq, *J* = 9.3, 6.9 Hz, OCH_2_CH_3_), 3.84 (1H, dq, *J* = 9.3, 7.2 Hz, OCH_2_CH_3_), 4.13 (1H, dd, *J* = 7.5, 5.1 Hz, 5-H), 5.42 (1H, dd, *J* = 4.5, 2.7 Hz, 7-H), 7.36 (2H, d, *J* = 8.4 Hz, Ar), 7.78 (2H, d, *J* = 8.4 Hz, Ar), 9.95 (1H, s, CHO) ppm; ^13^C NMR (75.5 MHz, CDCl_3_): δ = 14.8, 28.0, 28.8, 33.9, 35.0, 66.5, 88.4, 101.7, 128.1, 129.7, 134.8, 151.1, 151.2, 155.2, 162.7, 192.0 ppm.

##### *(5RS,7RS)*-*7*-*Ethoxy*-*5*-*(4*-*formylphenyl)*-*1,5,6,7*-*tetrahydro*-*1,3*-*dimethyl*-*2H*-*pyrano[2,3*-*d]pyrimidine*-*2,4(3H)*-*dione* (*trans***-8a**, C_18_H_20_N_2_O_5_)

Colorless crystals; mp: 168–170 °C; *R*_f_ = 0.29 (*t*-BuOMe); IR (powder): $\bar{\nu }$ = 2,951, 2,898, 2,823, 2,732, 1,698, 1,634, 1,574, 1,488, 1,169, 1,118, 1,043, 1,005 cm^−1^; ^1^H NMR (300 MHz, CDCl_3_): δ = 1.26 (3H, t, *J* = 7.2 Hz, OCH_2_CH_3_), 2.09 (1H, ddd, *J* = 13.8, 6.0, 2.4 Hz, 6-H), 2.27 (1H, ddd, *J* = 13.8, 7.2, 6.6 Hz, 6-H), 3.28 (3H, s, N-Me), 3.44 (3H, s, N-Me), 3.67 (1H, dq, *J* = 9.3, 6.9 Hz, OCH_2_CH_3_), 3.95 (1H, dq, *J* = 9.6, 7.2 Hz, OCH_2_CH_3_), 4.20 (1H, dd, *J* = 6.3, 6.0 Hz, 5-H), 5.24 (1H, dd, *J* = 6.9, 2.4 Hz, 7-H), 7.37 (2H, d, *J* = 8.1 Hz, Ar), 7.83 (2H, d, *J* = 8.4 Hz, Ar), 9.97 (1H, s, CHO) ppm; ^13^C NMR (75.5 MHz, CDCl_3_): δ = 15.0, 28.0, 28.7, 34.2, 35.9, 66.0, 88.3, 100.9, 127.9, 130.2, 135.2, 151.1, 151.2, 155.4, 162.0, 191.7 ppm.
